# COVID-19 Causing Hypotension in Frail Geriatric Hypertensive Patients?

**DOI:** 10.3390/medicina57060633

**Published:** 2021-06-18

**Authors:** Marek Koudelka, Eliška Sovová

**Affiliations:** 1 Department of Exercise Medicine and Cardiovascular Rehabilitation, Palacký University Olomouc and University Hospital Olomouc, 77520 Olomouc, Czech Republic; eliska.sovova@fnol.cz; 2 Department of Internal Medicine, FRANZISKUS SPITAL GmbH, Landstraßer Hauptstraße 4a, 1030 Vienna, Austria

**Keywords:** blood pressure, COVID-19, hypotension, hypertension, geriatric

## Abstract

Background: The association of coronavirus disease 2019 (COVID-19) with hypertension has been one of the frequently discussed topics in current studies since hypertension was identified as a risk factor for coronavirus disease. However, no studies seem to be focused on the BP (blood pressure) in patients with hypertension after COVID-19. Report: This report presents the cases of five frail geriatric patients (avg. age 78.3 (±6.4) years) with sarcopenia and controlled hypertension (office BP < 140 mmHg) who were diagnosed with SARS-CoV-2. Findings: Control ABPM performed after COVID-19 showed that these hypertensive patients were hypotensive and that the previously well-established therapy was suddenly too intensive for them. Conclusions: These findings suggest that BP control after COVID-19 is needed and that ABPM is, particularly in frail geriatric patients, by no means a luxury but a necessity.

## 1. Introduction

Coronavirus disease 2019 (COVID-19) is caused by severe acute respiratory syndrome coronavirus 2 (SARS-CoV-2). In many people, it has led to serious health complications and death. COVID case-fatality rates suggest that the individuals aged 60 years or older are most at risk. The highest case-fatality rate occurs in patients of 80 years and older [1]. A common problem in elderly individuals, reaching a prevalence as high as 60 to 80%, is hypertension, which has been previously shown as a risk factor for worse COVID-19 outcomes [2], as well as frailty [3], posing a higher mortality risk in frail geriatric patients.

According to studies, hypertension and cardiovascular disease are among common comorbidities in patients with COVID-19 increasing the likelihood of hospitalization and death. Ran et al. [4] suggest that higher BP (blood pressure) (not specific medication use) is an important independent risk factor for complications (e.g., heart failure) in hypertensive patients with COVID-19. Arterial hypertension is associated with more than twice the risk of suffering from severe forms of COVID-19 and, in those with cardiovascular disease, more than three times [2].

To the best of our knowledge, there is no study focusing on blood pressure (BP) control in hypertensive patients after COVID-19. In this report, we describe the decrease of BP values in five polymorbid frail geriatric hypertensive patients with sarcopenia after COVID-19. The objective of this case report is to inform about the necessity to control the effectiveness of BP treatment after COVID-19, preferably by ABPM.

## 2. Case Report

This case report presents five frail geriatric patients with sarcopenia treated for arterial hypertension for more than 5 years. These patients tested positive for COVID-19 (PCR tests). The age range of patients was 65–85 years and two of five patients were men. Basic clinical parameters of the five patients before and after COVID-19 are presented in Table 1, showing the worsening frailty and sarcopenia. To assess frailty, we used the frailty index for its complexity and precision (compared to, e.g., the Barthel test) since it considers physical and psychosocial aspects of frailty and cognitive functions. The frailty index was calculated for each patient as the number of deficits in a patient divided by all deficits considered (40 health deficit variables in our case) [5]. The frailty index ranges from 0.00 to 1.00, with a higher value indicating a worse/frailer status.

Patients had controlled hypertension before COVID-19, and their office BP was <140/90 mmHg (Table 2). The office BP measurements were performed according to the ESC/ESH guidelines [6], a standard bladder cuff was used, the BP was measured in a seated position and the measurements were performed using a regularly calibrated standard sphygmomanometer.

Twenty-four-hour ABPM was performed in patients during 2018–2019, i.e., less than a year before they tested positive for COVID-19 (PCR tests). ABPM showed satisfactory target values (Table 2). Patients also underwent clinical examinations, including heart rate and ECG measurements, before and after COVID-19; however, no difference was observed (values were within the normal range, and no anomaly or change was observed before or after COVID-19).

After COVID-19, their BP was monitored in the office, and well-controlled BP was shown in all patients (Table 2). Thereafter, we performed routine control of the effectiveness of the treatment using 24 h ABPM at the patient’s home following the currently valid guidelines [6] using the Mobil-O-Graph NG (same device, guidelines and other conditions as were used during ABPM before COVID-19) and detected hypotension in our patients (Table 2). BP measurements were taken every 20 min during the daytime and every 30 min at nighttime. Nighttime was defined as the time between 10 p.m. and 6 a.m. Target values were considered to be the following: mean BP (MBP) 130/80 mmHg over 24 h; MBP 135/85 mmHg during the daytime and MBP 120/70 mmHg during the nighttime [6]. To consider the ABPM successful, the record must provide a minimum of 20 valid daytime and 7 nighttime measurements, and at least 70% of the expected 24 h readings must be valid in compliance with current recommendations [6]. This ABPM was performed 3–6 months after the patient left the hospital, precisely in the following time interval: Patient No. 1: 4 months; Patient No. 2: 5 months; Patient No. 3: 3 months; Patient No. 4: 5 months; Patient No. 5: 6 months.

There was no change in the lifestyle, treatment regimens or medication adjustment since the last BP compensation control performed by ABPM during 2018–2019. COVID-19 was not treated with drugs influencing BP (only symptomatic therapy—fever, productive cough; no corticoids).

As for hypertension therapy, patients were treated with ACE inhibitors, angiotensin receptor blockers (ARBs), calcium channel blockers (CCBs), diuretics and beta-blockers. Their distribution can be seen in Table 3.

There was no change in antihypertensive medication between the two ABPMs. Before and after COVID-19, preparation and usage of medication were in the control of families or carers. During the hospitalization, when patients had a fever, they were taken off diuretics and an adequate intake of liquids was ensured by IV rehydration. When their hospitalization finished, they started to use diuretics regularly again as they did before the fever. No patients had contraindications. During the febrile state, the intermittent deterioration of chronic kidney disease in the context of pre-renal failure was observed. However, after careful hydration of patients, the renal functions were stable and within the same range [7]. We did not observe any polyureic phase in the patients.

Patients had the following medical history: ischemic heart disease (2/5), stroke/TIA (1/5), diabetes mellitus and prediabetes (IGT) (2/5), atrial fibrillation (1/5), obstructive pulmonary disease (1/5) and chronic kidney disease (3/5).

There was no difference in BP in smokers, ex-smokers and non-smokers.

Patients 1–5 were hospitalized with COVID-19 for 36, 28, 38, 43 and 45 days, respectively. During hospitalization, Patients 1 and 3 required supplemental oxygen but did not require oxygen delivery through a high-flow device. They had dexamethasone therapy. Patient 2 had mild to moderate COVID-19 (absence of viral pneumonia and hypoxia) and was provided supportive care (as is recommended also by the Panel [8]). Patients 4 and 5 required delivery of oxygen through a high-flow device or noninvasive ventilation.

Our patients did not show any signs of dehydration, hypovolemia or hyperhydration/overhydration during the clinical examination. We performed pro-BNP at the beginning of each hospitalization, as usual, and the values were within standard range/limits in our patients. Patients’ nutrition was standard and was not significantly modified as per quality—patients consumed the same kind of food before and after COVID (the salt intake was not decreased, although recommended following the DASH). After COVID, we observed loss of weight that can be attributed to the reduction of muscle mass caused by hypomobility and to disease-related malnutrition (DRM), which we monitored in a comprehensive geriatric assessment (CGA).

**Figure 1:**
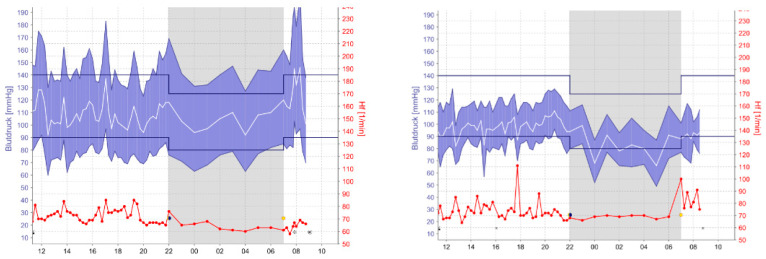
BP (blood pressure) values of Patient 2 before and after COVID-19.

As shown in the ABPM (Figure 1), the detected BP values of patient 2 decreased appreciably. This phenomenon was observed in all five patients. To avoid potential complications (e.g., falls, hypoperfusion of target organs) and CV risks related to hypotension, the therapy had to be adjusted, and the medication intake was reduced for each patient.

We are not aware of any factors that could contribute to BP lowering in these five patients. After COVID-19, the patients faced common post-COVID complications such as long-COVID fatigue, faintness, low performance/productivity and sometimes vertigo.

## 3. Discussion

To the best of our knowledge, no study has focused on the BP in patients with hypertension after they recovered from COVID-19, especially not in frail geriatric patients with sarcopenia. During the BP measuring in the office after COVID-19, the patients were normotensive, and the therapy seemed to be set well. Nevertheless, after routine control of the effectiveness of the treatment (24 h ABPM), we detected hypotension in our patients. This suggests we cannot only rely on the office BP measurement since one-off measurements may not accurately reflect the BP of a patient, but we should use the 24 h ABPM as a complementary measurement (as suggested by ESC/ESH Guidelines [6]), if not as a key tool for correct treatment management. Even though 2018 ESC/ESH Guidelines do not provide formal ABPM BP targets for treated patients, they note that a target office systolic blood pressure (SBP) of 130 mmHg might correspond to a slightly lower mean 24 h SBP (i.e., approximately 125 mmHg) and that the difference between office BP and ambulatory BP values diminishes and becomes negligible at an SBP of approximately 120 mmHg.

According to 2018 ESC/ESH Guidelines, the desired SBP target range for all patients aged >65 years is 130–139 mmHg and diastolic blood pressure (DBP) of <80 mmHg if tolerated, and treated SBP values of <130 mmHg should be avoided [6].

Since ABPM in our patients showed hypotension (both SBP and DBP were much lower), the therapy and medication intake had to be adjusted to avoid potential risks because “the lower the better” does not seem to be valid for elderly patients. Results of the PARTAGE study show that frail patients (>80 years) with very low SBP (<130 mmHg) who are taking multiple antihypertensives (two or more) have an increased risk of mortality [9]. Similarly, The English Longitudinal Study of Ageing (ELSA) study focusing on octogenarians also showed an increase in mortality rates appearing at SBP of <110 mmHg (not only when SBP ≥ 170 mmHg) [10]. A post hoc analysis of The Systolic Blood Pressure Intervention Trial(SPRING) demonstrated a clear J-shaped relationship between the effect of intensive BP control and the risk of cardiovascular disease (CVD); nevertheless, the J-shaped relationships between both DBP and SBP and main cardiovascular outcomes seem to concern patients with an extensive atherosclerotic burden according to the Hypertension in the Very Elderly Trial (HYVET) and the International Verapamil SR Trandolapril Study (INVEST) [11].

Current findings from Sheppard et al. [12] show that patients with BP < 130/80 mm Hg had higher odds of COVID-19 death than patients with BP 140/90–159/99 mm Hg. BP values 130/80–139/89 mmHg and BP ≥ 160/100 mmHg were not associated with COVID-19-related death. They suggest that worse COVID-19 outcomes associated with BP < 130/80 mm Hg might possibly be due to more advanced atherosclerosis and a higher prevalence of target organ damage. Their findings support that the SBP should not be lowered to <130/80 mm Hg (in accordance with ESC/ESH Guidelines [6]) as mentioned above.

ABPM measuring was performed 3–6 months after the patients left the hospital; therefore, we assume BP-lowering to be a tardive effect. Current guidelines [6] state that ABPM may be repeated in 1- or 2-year intervals. The ABPM performed after COVID-19 was the regular ABPM check because office BP values seemed to be fine and there was no suspicion of white-coat, masked or nocturnal hypertension, which would lead us to proceed with ABPM earlier. The question of how BP values evolve in these patients remains to be answered in the future since the mechanism is still to be clarified. Next ABPM measuring is planned in 3–6 months. Currently, the patient performs HBPM and will bring the protocol (diary with the BP values) to the next appointment. Should guidelines mention now that more frequent repetitions of ABPM are required in some patients?

To assess frailty, we used the frailty index for its complexity and precision. Nevertheless, frailty is a complex phenomenon with a multifactorial etiology; therefore, there is no diagnostic standard, and the aging phenotypes and gender dimorphism should be taken into account [13,14]. Frailty includes a broader spectrum of deficits (e.g., comorbidities, cognitive and mood decline) and could predict the susceptibility to adverse outcomes with higher discrimination than a definition limited only to physical dysfunction [15]. As showed by Corrao et al. [14], gender differences are known for diseases in terms of age distribution and impact of risk factors, clinical presentation and outcomes—with women often having worse prognoses than men, including in terms of depression, physical function decline and poor quality of life. For instance, feeling lonely has been associated with higher age-related increases of systolic BP [16]. Since we present only five patients (two men, three women), it is a very small sample to observe great differences. However, we are aware that care for elderly people must be personalized in order to improve their quality of life. Still, we face the same problem as Marcucci et al. [13], who pointed out the need for a better implementation and integration of social and health care assistance for these patients outside the hospital in order to improve the post-acute phase after hospital discharge, prevent rehospitalization and delay the progression of frailty.

As a consequence of COVID-19, patients are facing disruption of muscle function, and the total amount of muscle mass is reduced (sarcopenia) due to hypomobility and disease-related malnutrition. Their disability progresses, and they are dealing with even more self-care limitations in activities of daily living (ADL) and instrumental activities of daily living (iADL). Geriatric hypomobility syndrome, deconditioning and muscle weakness contribute to the BP lowering as mentioned for example in the study of Joyner and Masuki [17]. The host of bed rest deconditioning studies suggest that after periods of bed rest deconditioning, there is a reduction in heart volume (cardiac atrophy), a reduction in blood volume and marked tachycardic responses to standing or upright tilting which can also include unusually high levels of blood pressure variability [17].

Nevertheless, we expect there is also another factor or mechanism influencing the BP. We presume that this decrease in BP is associated with ACE (Angiotensin-converting-enzyme) inhibitors and beta-blockers, which are currently discussed as drugs influencing the risk of COVID-19 and its severity. At the beginning of the pandemic, using ACE inhibitors was hypothesized to present an increased risk of developing COVID-19 disease with a severe course due to the presumed higher expression of the ACE2 enzyme in the lung cells. The SARS-CoV-2 virus uses the enzyme ACE2 to enter cells (especially in the lungs). Based on the assumption that hypertensive patients taking ACE inhibitors have increased expression of this enzyme, some authors have suggested that when these patients are infected with the SARS-CoV-2 virus, the virus will extensively enter primarily into the lungs and that this treatment means an increased risk of developing COVID-19 and a more severe course. However, as Hippisley-Cox et al. [18] showed, treatment did not increase but even reduced the risk of the patient developing COVID-19 (HR 0.71, 95% CI 0.67–0.74). Furthermore, the study showed that in patients with COVID-19, the use of ACE inhibitors did not increase the severity of the course; i.e., the risk of hospitalization in the ICU was not higher.

SARS-CoV-1 and SARS-CoV-2, which have been responsible for the SARS epidemic and the COVID-19 pandemic, respectively, interface with the renin–angiotensin–aldosterone system (RAAS) through ACE2, an enzyme that modulates the effects of the RAAS but is also the primary receptor for both SARS viruses [19]. The interaction between the SARS viruses and ACE2 may be one determinant of BP decrease in our patients.

SARS-CoV-2 binds and degrades ACE2, thereby potentially reducing its counter-regulatory effects [20]. If ACE2 effects are reduced and additionally inhibited also by antihypertensives, then the BP values are decreasing, which would potentially explain the phenomenon observed in our patients. Post-COVID-19 effects and complications are present in some patients for several months. ACE2 might have still been inhibited by SARS-CoV-2 even in the time we performed ABPM after COVID-19; however, what if, and this might be a very daring hypothesis, SARS-CoV-2 reduces not only its counter-regulatory effects but also the number of ACE receptors? Since there would be less renin–angiotensin–aldosterone system (RAAS) activity, the BP would also be lower. Nevertheless, this is only a hypothesis and would need to be confirmed by research.

Our patients were also taking beta-blockers. Data of Reynolds et al. [21] suggested a modestly lower likelihood of a positive test for COVID-19 among patients taking beta-blockers that was of marginal significance in an analysis that included all matched patients. This finding could be attributable to the effects of beta-blockers on the expression or presentation at the cell surface of ACE2, the viral receptor for SARS-CoV-2, or residual confounding in the observational study design. However, further studies are required. Because beta-blockers, ACE inhibitors and ARBs act at different points in the RAAS, their effects on the risk of COVID-19 or the severity of COVID-19 could potentially differ [21]. Therefore, their impact on the BP decrease might also be different.

Some of our patients are ex-smokers and smokers. Smoking may cause increased ACE2 mRNA expression in human lung much as ACE inhibitors or ARBs are believed to, suggesting a possible common protective mechanism for severe COVID-19 disease [22], and possibly influence the BP values.

Hypotension after COVID-19 may be presented in a lower number of hypertensive patients, but we would like to bring attention to it, especially when hypertension occurs in frail geriatric patients who have a higher mortality risk and are prone to the progression of cognitive frailty. Low BP, both diastolic and systolic, in the populations of the elderly is correlated with worse cognitive performance. Hypotension and excessive treatment of hypertension may induce cerebral hypoperfusion, ischemia (the perfusion pressure distal to the epicardial coronary artery stenosis) and hypoxia, leading to neurodegenerative processes that speed up the clinical manifestations of cognitive impairment and dementia [23].

Our case report shows that there might be an increased risk which hypertensive patients may face after COVID-19: overmedication. It also highlights the importance of ABPM. Changes in BP values which are often not detected by common office BP measuring but are revealed by ABPM suggest its key role in the therapy and treatment management of hypertensive patients.

### Implications for Clinical Practice

These findings suggest that BP control after COVID-19 is needed and that ABPM is by no means a luxury but rather a necessity, especially in frail geriatric patients. Therefore, it is recommended to proceed with a more profound BP check than office BP measurement, particularly in frail geriatric patients.

## Figures and Tables

**Table 1:** Basic clinical parameters before and after COVID-19 (Coronavirus disease 2019).

	Sex	Smokers	Diabetes	Age (years)		Weight (kg)		Height (cm)		Body Mass Index		Frailty Index (Points)		Dominant Hand Grip Strength (kg)	
				before COVID-19	after COVID-19	before COVID-19	after COVID-19	before COVID-19	after COVID-19	before COVID-19	after COVID-19	before COVID-19	after COVID-19	before COVID-19	after COVID-19
**Pat. No. 1**	Female	Never smoked	Yes	75	75	75	70	168	167	27	25	0.58	0.65	15	13
**Pat. No. 2**	Female	Ex-smoker	Yes	81	83	80	75	180	180	25	23	0.48	0.58	14	10
**Pat. No. 3**	Male	Ex-smoker	No	82	82	68	80	170	170	23	29	0.40	0.55	22	16
**Pat. No. 4**	Female	Active smoker	No	68	68	76	52	169	169	27	21	0.45	0.53	13	10
**Pat. No. 5**	Male	Never smoked	No	85	86	56	51	178	178	17	16	0.38	0.60	26	14

**Table 2:** BP results for each patient.

	Office BP				24 h ABPM (MBP)											
	before COVID-19		after COVID-19		before COVID-19		after COVID-19		before COVID-19		after COVID-19		before COVID-19		after COVID-19	
	SBP	DBP	SBP	DBP	24 h		24 h		Daytime		Daytime		Nighttime		Nighttime	
					SBP	DBP	SBP	DBP	SBP	DBP	SBP	DBP	SBP	DBP	SBP	DBP
**Pat. No. 1**	125	80	120	80	130	77	115	67	132	78	116	67	123	70	111	64
**Pat. No. 2**	120	85	130	90	148	78	114	79	149	48	116	81	142	73	102	68
**Pat. No. 3**	135	75	135	80	121	76	93	58	125	79	92	58	95	60	94	57
**Pat. No. 4**	135	85	130	85	131	72	108	67	131	73	109	68	126	68	97	59
**Pat. No. 5**	130	80	125	75	121	74	114	65	120	74	115	66	128	75	112	59

BP = blood pressure, ABPM = ambulatory blood pressure monitoring, MBP = mean blood pressure, SBP = systolic blood pressure, DBP = diastolic blood pressure.

**Table 3:** Antihypertensive medication.

	Angiotensin-Converting-Enzyme (ACE) Inhibitors	Angiotensin Receptor Blockers (ARBs)	Calcium Channel Blockers (CCBs)	Diuretics	Beta-Blockers	Dual Therapy	Triple Therapy	Quadruple Therapy

**Pat. No. 1**	√		√	√			√	
**Pat. No. 2**		√	√			√		
**Pat. No. 3**	√				√	√		
**Pat. No. 4**	√		√	√	√			√
**Pat. No. 5**		√		√		√		

## Data Availability

The datasets used or analyzed during the current study are available from the corresponding author on reasonable request.
